# Incidence of childhood cancer in Namibia: the need for registries in Africa

**DOI:** 10.11604/pamj.2014.17.191.3830

**Published:** 2014-03-12

**Authors:** Daniela Cristina Stefan, Bjorn Baadjes, Mariana Kruger

**Affiliations:** 1 Department of Paediatrics and Child Health, Stellenbosch University, Cape Town, South Africa

**Keywords:** Childhood cancer, registry, Namibia, incidence

## Abstract

**Introduction:**

Childhood cancer is rare and comprises only 1% of all cancers. The current incidence of childhood cancer in Namibia, as in many other African countries, is not known. The aim of this research was to assess the paediatric cancer incidence between 2003-2010 at Windhoek Central Hospital, the only pediatric oncology-referring centre in Namibia and to compare with the previous calculated incidence in the country 20 years ago.

**Methods:**

A retrospective, descriptive review of the paediatric oncology cases presenting to Windhoek Central Hospital between 2003 and 2010 was undertaken, and data regarding age, sex, cancer type, area of residence were extrapolated. In this study due to the appearance of the HIV epidemic, an HIV incidence was also calculated.

**Results:**

The incidence rate of all paediatric recorded cancers was 29.4 per million. Leukaemias (22.5%) and retinoblastomas (16.2%) were the most common tumours, with renal tumours, soft tissue sarcomas and lymphomas following in frequency. HIV incidence of children with malignancy was 6.8%.

**Conclusion:**

The incidence rates of cancers in this study are remarkably lower compared to a similar study done in the country 20 years ago. Many cancers are still not diagnosed or reported, and others are not treated in the country. The institution of a “twinning programme” between the paediatric haematological/oncological departments in Windhoek and Tygerberg Hospital in Cape Town, South Africa, will contribute to improvement of childhood cancer cases. This twinning programme includes the formation of a cancer registry.

## Introduction

Childhood cancer is rare and comprises only 1% of all cancers [1]. However, worldwide there are more than 200,000 new cases of childhood cancer per year, and more than 70% of these occur in the developing world. After accidents cancer is one of the most frequent causes of death in children in Western countries [2], while in Africa it is not even ranked among the 10 most common causes of death. Infections, nutritional disease, HIV and tuberculosis remain the most important paediatric health problems in developing countries [3].

In developed countries up to 80% of children with malignancies can be cured [2], but unfortunately most children live in developing countries, where the cure rate is much lower [4]. In first world countries more than 70-80% of children with cancer become long-term survivors [2]. For some childhood cancers 5-year survival rates approach 95% [5]. Unfortunately the cure rate for children in developing countries does not reflect the same success.

Most African countries do not have a cancer registry, so the real incidence and types of cancer and survival are not known [6, 7].

A previous study on childhood cancer in Namibia by Wessels in 1988 showed the epidemiology of tumours in the country [8]. A tumour registry was subsequently established, but was discontinued in the late 1990s.

Due to a long standing relationship between the 2 neighboring countries, a twinning program has been established between Windhoek Central Hospital in Namibia and Tygerberg Hospital in Cape Town South Africa in 2009 with the objectives of building up capacity in teaching and training of pediatric oncology in Namibia, offering scientific and academic support regarding the treatment of patients locally, as well as encouraging the formation of a Namibian parent support group and training of auxiliary team members in oncology (pharmacists, social workers, dieticians, etc).

In the context of the newly formed twinning program between the 2 units and also with an estimated 70% of total pediatric cancers expected to occur in developing countries, the aim of this research was to analyze the incidence of paediatric cancers presenting to the only referring hospital in Namibia, the Windhoek Central Hospital, between January 2003 and December 2010.

The trends of incidence as well as outcome were assessed and compared with previous results [8] published 20 years ago. Age, different malignancy, stages at presentation, differences between the sexes and ethnic groups, and outcomes were assessed. In the context of an HIV epidemic this study calculated an HIV incidence of all children diagnosed with a malignancy.

## Methods

An audit of paediatric cancers presenting to the Windhoek Central Hospital between January 2003 and December 2010 was performed. The data captured for the study consisted of information gathered from patients’ medical records at the paediatric oncology ward at the Windhoek Central Hospital.

All children below the age of 15 years diagnosed with cancer who presented to Windhoek Central Hospital between 2003-2010 were included. Date of birth and age at diagnosis, sex and region of residence were among the variables evaluated in the study. Data included the type of cancer diagnosed and, where possible, the cancer stage and eventual outcome of the patient. Incidence of HIV occurrence was also evaluated. However, a significant amount of information from the files was incomplete, and several patients were also subsequently lost to follow up.

All malignancies were confirmed histologically by a pathologist and were reviewed by a tumor board. No patients were excluded from the study.

A statistician was consulted to assist with the research. Incidence and frequency rates of the various cancers were performed. Mean age at diagnosis for each tumour was calculated, and differences between the two sexes were observed. Incidence of children with cancer and concomitant HIV was noted. Comparison with a similar study done by Wessels, et al [8] in 1988 was also performed.

**Ethical approval**: Ethical approval for this study was obtained from the Health Research Ethics Committee of the University of Stellenbosch; ethics reference number N10/11/384. The superintendent of Windhoek Central Hospital also gave approval to the collection of data from records and completion of the study.

## Results

Between January 2003 and December 2010, out of 191 patients admitted to the Windhoek Central Hospital with cancer, leukaemias and retinoblastomas occurred most frequently, with frequency rates of 22.5% and 16.2%, respectively (Table 1). Thereafter, renal tumours, soft tissue sarcomas and lymphomas occurred. Most of the leukaemia patients had acute lymphoblastic leukaemia (ALL 88.4%).

**Table 1:** Total number, relative frequency and incidence rates of cancers presenting to Windhoek State Hospital 2003-2010

Cancer type	Number of cases	Relative frequency (%)	Incidence rate (per million)
Leukaemias	43	22.5	6.6
Lymphomas	23	12.0	3.5
Brain and spinal tumours	10	5.2	1.5
Sympathetic nervous system tumours	12	6.3	1.8
Retinoblastomas	31	16.2	4.8
Renal tumours	26	13.6	4.0
Hepatic tumours	4	2.1	0.6
Malignant bone tumours	5	2.6	0.8
Soft tissue sarcomas	26	13.6	4.0
Germ cell and gonadal neoplasms	8	4.2	1.2
Epithelial neoplasms	2	1.0	0.3
Other neoplasms	1	0.5	0.2
Total	191	100	29.4

**Figure 1:**
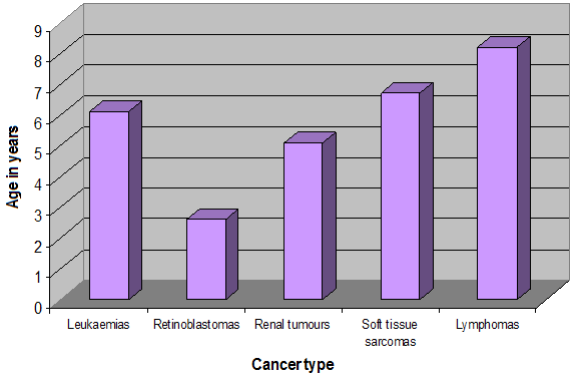
Mean age (in years) at diagnosis of most prevalent cancers

Lymphomas had a frequency rate of 12%, with a mean age of 8 years (Figure 1), brain tumours occurred at a frequency rate of 5.2%, while malignant bone tumours of only 2.6%.

**Figure 2:**
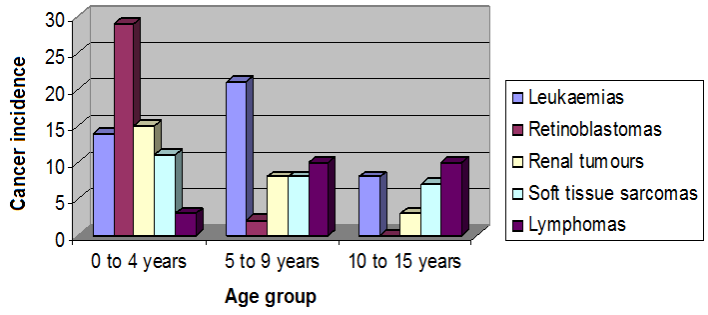
Cancer incidences at 0 to 4 years, 5 to 9 years and 10 to 15 years

The mean age at diagnosis for all cancer types was 5 years and 3 months (Figure 2) and was similar to the international data (leukaemia 6 years and 1 month, retinoblastomas 2 years, 7 months, renal tumours and soft tissue sarcomas 5 years, 1 month, and 6 years, 8 months, respectively).

Leukaemias and lymphomas occurred in higher frequency in boys (13.6% and 8.9%, respectively) compared to girls (8.9% and 3.1%, respectively) and retinoblastomas were also more prevalent in boys (9.9% compared to 6.3% in girls).

The adult prevalence rate of HIV in Namibia was estimated at 13.1% in 2009, [9]. During the 2010/2011 fiscal year the prevalence of HIV in children below 15 years was estimated at 2.11%, with roughly 17500 infected, [10]. The frequency of HIV in the oncology unit increased over the study period, as HIV testing became compulsory in 2008. In 2003 and 2005 only one patient was diagnosed with HIV, with concomitant Kaposi sarcoma. In 2010 six patients were diagnosed with HIV – four of these had Kaposi sarcoma whilst two others had Burkitt lymphoma.

Trends of incidence confirmed leukaemias as the most common malignancy in Namibian children. Whilst retinoblastomas and lymphomas were more common in the first 4 years, renal tumours and soft tissue sarcomas occurred more frequently between 2007 and 2010. Wilms tumour was the predominant renal tumour during this period. Brain tumours presented to the Windhoek Central Hospital paediatric oncology unit with a much higher frequency towards the latter part of the study period.

Cancer stage and treatment modalities offered, as well as differences between ethnic groups and outcome of individual patients could unfortunately not be assessed as recorded data was insufficient and a large proportion of patients was lost to follow up. Prior to the intended implementation of a twinning partnership between the paediatric haematology/oncology department at Tygerberg Hospital and the paediatric oncology unit at the Windhoek Central Hospital, the overall survival of childhood cancers in the country was estimated at only 17% [11].

## Discussion

Over the past decades there has been a lot of research into paediatric oncology. The majority of this research, however, has been done in developed countries [6]. The United States’ National Cancer Institute, for example, has the Surveillance, Epidemiology and End Results (SEER) Programme, which collects data from all over the country, and extrapolates this to determine national childhood cancer data, [12].

However, little or no research in the cancer field has been performed in Namibia or in many African countries where most cancers are expected to occur in children.

According to SEER data, in children aged 0 to 14 years leukaemias are the commonest cancer with an incidence rate of 50.5 per million, followed by brain and spinal tumours at 37.3 per million and lymphomas at 15 per million (2001 to 2004). 5-year survival rates for childhood cancers reach 78% (80% for leukaemias, 70% for brain and spinal tumours and 87% for lymphomas). Retinoblastoma 5-year survival rates reach as high as 97% (1996 to 2003), [13].

After accidents, cancer is the leading cause of death in children around the world [14]. Over the past 20 years the advent of HIV has overshadowed childhood cancer in sub-Saharan Africa. However, despite this, cancer still remains an important killer of children in Southern Africa [15], and indeed, the infection with HIV increases the association with malignancies [16–18].

Namibia is a young, developing country of 23 years. The population of Namibia for the study period is estimated at roughly 2.17 million. Children below 15 years of age made up about 33.4% of this value (roughly 723,000). This information was gathered from the CIA World Factbook, and is considered the most accurate at this time, [9]. The last census conducted in Namibia was in 2001, with another being performed in 2011. The bulk of the inhabitants reside in the northernmost and central parts of the country. It has a well-established medical infrastructure, with the majority of serious illnesses being referred to the State Hospital Complex in the capital city, Windhoek, which is situated in the central part of the country. In particular, children with cancer from all regions within the country are admitted in a special dedicated ward with 20 beds at the Windhoek Central Hospital. The incidence of childhood cancer in Namibia, as in many other African countries, is not known.

The incidence rate of all cancers in the study was 29.4 per million. This rate is significantly lower than the incidences reported in other countries [2], and indeed lower than that recorded in the same population 20 years previously [8]. The possible reasons for the lower incidence could be related to the lack of a registry, under diagnosis and under reporting. Although the medical infrastructure is quite well established, a vast majority of the population is still settled in rural areas, and many do not seek the attention of western medicine when ill. Also, a lack of experienced health care workers and concomitant infectious diseases, which occur so commonly in the country, add to the misdiagnosis of cancer [19].

Previously, paediatric patients with cancer in Namibia were referred to Tygerberg Hospital for treatment and their data was captured in a tumour registry which was established in 1983 [8]. However, this relationship between the two hospitals no longer existed and the tumour registry was discontinued after 1992, with inadequate capturing of data following.

Although most childhood cancers or suspected cancers in Namibia are referred to the Windhoek Central Hospital, there are some children who are treated elsewhere. As there are no paediatric oncologists and no other cancer centres in Namibia, patients with medical insurance are treated by private paediatricians or general practitioners, or are referred to South Africa for further management.

Furthermore, most children with brain and spinal tumours are managed by the radiotherapy specialists and are not referred to the paediatric oncology ward at Windhoek Central Hospital. Kaposi sarcoma cases are treated in the HIV clinic, and are rarely referred to the oncology unit. These factors all contribute to the seemingly very low incidence rate of childhood cancer in the study.

Leukaemias occurred most frequently at Windhoek Central Hospital during the 8 year study period. Incidence of leukaemia doubled in this period (22.5%) compared to 11.6% incidence reported by Wessels [8] in 1988. Retinoblastomas had the second highest frequency rate, and also increased to 16.2% from 10.5% in the previous study.

As was the case in the study 20 years earlier, renal tumours made up 13.6% of the cancer types presenting to Windhoek Central Hospital. This is comparable to the incidence in Southern Africa [3] and is significantly higher than in developed countries where it represents less than 7% of cancers in children [2, 13]. Soft tissue sarcomas also comprised 13.6% of the cancers.

Retinoblastomas and renal tumours are cancers, which can potentially be diagnosed and treated early, occur at higher frequency rates at Windhoek Central Hospital. An awareness of these cancers will improve eventual survival rates.

The incidence of brain and spinal tumours is significantly low in this study, with brain tumours occurring at a rate of 5.2%, compared to 17.8% in the study by Wessels [8]. In other countries it is the second commonest cancer, following leukaemias [2, 12, 13, 20, 21]. Along with other adult tumours, these cancers are primarily treated by radiotherapy specialists, and are thus not referred to the children's oncology ward at Windhoek Central Hospital. Their numbers in this study are therefore understandably low, with severe underestimation of their likely incidence in the population of Namibia.

Bone tumours were also under diagnosed: previously occurring at a frequency rate of 8.1%, these tumours now made up only 2.6%. The cause of this discrepancy could not be ascertained; hypothesizing that exclusively the orthopaedic surgeons treated them and were not referred to the pediatric oncology team.

In 2009 it was estimated that 180,000 of the Namibian population was HIV positive, [9]. Although the relationship between cancer and HIV is well established, the number of children found to also be HIV positive in this study was small. The reason for the infrequent numbers of HIV positive children in the study is most likely under diagnosis due to lack of testing. The frequency is noted to be increasing towards the latter years in the study, where HIV testing is likely to have been more readily performed. The HIV incidence of children diagnosed with a malignancy was estimated at 6.8%. Routine testing for HIV infection of all patients presenting with cancer has been introduced since 2009.

This study reports an increase in the incidence of leukemia, a constant high previously reported incidence of nephroblastoma and retinoblastoma but with an overall incidence low, compared to published data in developed countries.

### Limitations of the study

The study has some significant limitations. It is a retrospective study relying on documentation of cases presenting to the Windhoek Central Hospital. A lot of the data in the folders was incomplete and many patients were lost to follow up, establishing the cancer stage and assessment of overall outcomes impossible. Although Windhoek Central Hospital is the only recognized State hospital to offer treatment to Namibian children with cancer, many patients with medical insurance are still treated privately or referred to South Africa. Brain and bone tumours and Kaposi sarcomas were treated by other disciplines. Childhood cancers are severely under diagnosed in the country.

### The way forward

The need for registries in Africa remains a priority as the incidence of cancers remains unknown in most countries on the continent. Globocan provides only estimates of the total number of cancers and not a real calculation of the burden of the disease.

Along with the capture of data for this study, a tumour registry form was designed specifically for the Namibian population. It is intended that the use of this form to document data on individual patients’ cancer information, will eventually build up a cancer registry for the country. This information can also be collected at institutions other than the Windhoek Central Hospital, but stored at a central database. The tumour registry will prove invaluable to further research studies, and monitoring of childhood cancer trends in Namibia. Implementing an awareness programme to help detect and diagnose children with cancer early will improve the outcome of these children [19].

Moreover, the institution of a twinning programme between the oncology units at the Windhoek Central Hospital and Tygerberg Hospital aims at improving the diagnosis and treatment of childhood cancer; increase awareness, collection of data and a functional, complete tumor registry.

WHO has defined guidelines for national cancer control programms [22]. However, along with several other African countries [7], Namibia does not have an established national cancer control programme as defined by these guidelines. The burden of paediatric cancer in the country is unascertained presently.

An important priority in the care of children with cancer is early diagnosis and treatment, as well as regular follow up [23]. The establishment of well-functioning national paediatric cancer centres should also be a priority for governments [23]. These cancer centres would require adequate infrastructure for diagnosis, chemotherapy, radiotherapy, surgery and palliative care as well as trained paediatric oncologists and nurses.

With limited resources and lack of experienced personnel available in developing countries like Namibia, the care of children with cancer is compromised [24]. A twinning approach between developed and developing areas has gained success around the world [15], and it is planned to create such a partnership between the paediatric oncology unit at Windhoek Central Hospital and the Department of Paediatric Oncology at Tygerberg Hospital in South Africa.

## Conclusion

A global united effort of all those involved in the care of African children with cancer to establish and contribute to a cancer registry will give the much needed information required to plan further for the improvement of care and survival of our patients.
